# Analysis of retinal nerve fiber layer birefringence in patients with glaucoma and diabetic retinopathy by polarization sensitive OCT

**DOI:** 10.1364/BOE.402475

**Published:** 2020-09-10

**Authors:** Sylvia Desissaire, Andreas Pollreisz, Aleksandra Sedova, Dorottya Hajdu, Felix Datlinger, Stefan Steiner, Clemens Vass, Florian Schwarzhans, Georg Fischer, Michael Pircher, Ursula Schmidt-Erfurth, Christoph K. Hitzenberger

**Affiliations:** 1 Center for Medical Physics and Biomedical Engineering, Medical University of Vienna, Vienna, 1090, Austria; 2 Department of Ophthalmology and Optometry, Medical University of Vienna, Vienna, 1090, Austria; 3 Institute of Medical Information Management, Medical University of Vienna, Vienna, 1090, Austria

## Abstract

The retinal nerve fiber layer (RNFL) is a fibrous tissue that shows form birefringence. This optical tissue property is related to the microstructure of the nerve fiber axons that carry electrical signals from the retina to the brain. Ocular diseases that are known to cause neurologic changes, like glaucoma or diabetic retinopathy (DR), might alter the birefringence of the RNFL, which could be used for diagnostic purposes. In this pilot study, we used a state-of-the-art polarization sensitive optical coherence tomography (PS-OCT) system with an integrated retinal tracker to analyze the RNFL birefringence in patients with glaucoma, DR, and in age-matched healthy controls. We recorded 3D PS-OCT raster scans of the optic nerve head area and high-quality averaged circumpapillary PS-OCT scans, from which RNFL thickness, retardation and birefringence were derived. The precision of birefringence measurements was 0.005°/µm. As compared to healthy controls, glaucoma patients showed a slightly reduced birefringence (0.129 vs. 0.135°/µm), although not statistically significant. The DR patients, however, showed a stronger reduction of RNFL birefringence (0.103 vs. 0.135°/µm) which was highly significant. This result might open new avenues into early diagnosis of DR and related neurologic changes.

## 1. Introduction

Glaucoma and diabetic retinopathy (DR) are among the most widespread vision threatening diseases affecting the working age population worldwide [1,2]. Glaucoma is a heterogeneous group of diseases resulting in damage to the optic nerve, frequently associated with a high intraocular pressure. It first affects the peripheral vision and may cause blurred vision over time. In the early stage of the disease, diagnostics can be done by looking at changes in the appearance of the optic nerve head (ONH) and at visual field defects [3]. DR is the most common microvascular complication of diabetes causing a blurred and distorted vision. It is usually diagnosed with fundus examination where specific groups of lesions such as micro-aneurysms or retinal hemorrhages can be identified [4,5]. For glaucoma as for DR, early diagnostics and monitoring the development of the disease are essential in order to prevent vision loss.

Optical coherence tomography (OCT) is widely used in the clinic to analyze retinal diseases [6,7]. It provides high resolution images of retinal damages and allows following their evolution over time. In the case of glaucoma, OCT proved to be an advantageous method for the analysis of retinal ganglion cell (RGC) loss and of determining the thinning of the RNFL [8], which is a fibrous tissue consisting of the axons of the RGCs. RNFL thinning, as typically seen on peripapillary scans, is a particularly interesting parameter to discriminate between healthy and glaucomatous eyes [9,10]. Many studies report on the comparison of RNFL thickness to normative values for the diagnosis of glaucoma [11–13]. The inter-individual variability of RNFL thickness is, however, still a major obstacle for early and accurate glaucoma diagnostic [14,15].

In the case of DR, OCT offers the possibility to study the development of specific lesions such as macular edema [16,17]. OCT angiography (OCTA) is also commonly used to investigate the changes in vasculature occurring in the different plexuses [18]. While diagnostics is mainly based on the identification of vascular changes, it is however still unclear if they appear first in the development of the disease, or rather simultaneously or consecutively to neurologic changes. In parallel to the work done on vascular changes, studies were carried out to analyze changes in retinal nerve fiber layer (RNFL) thickness in diabetic patients, diagnosed with and without DR [19–21]. However, the outcomes of these studies are somewhat controversial.

While changes in the RNFL thickness can be investigated using a standard OCT system, other methods exploit the birefringence property of the RNFL. A first method that utilized the birefringence for RNFL analysis, and that found widespread clinical application, was scanning laser polarimetry (SLP) [22–25]. It measures the retardance caused by the RNFL and calculates the RNFL thickness by dividing the retardance by the birefringence, which was assumed to be constant (independent of RNFL position or disease state) [26]. However, this method has some shortcomings: it only gives access to 2D information and is prone to artifacts caused by the birefringence of other ocular layers like the sclera [27]. Moreover, it has been shown that RNFL birefringence is higher in the superior and inferior areas as compared to the temporal and nasal ones [28–30]. In addition, experiments in non-human primates, using a combination of SLP (retardance) and OCT (thickness) measurements, have shown that RNFL birefringence is reduced in experimental glaucoma prior to any thickness change of the tissue [31], likely caused by changes in the structure and/or composition of the axons of which it consists [32,33]. Based on this finding, birefringence analysis might provide early information on structural damage of the RNFL.

Polarization sensitive (PS-) OCT, a functional extension of OCT, allows retrieving the polarization information over a 3D volume of the retina [34–36]. Thereby, PS-OCT can differentiate polarization preserving, birefringent, and depolarizing tissue. Moreover, the 3D information can be used to calculate tissue birefringence from the measured thickness and the retardation introduced into the sampling beam by the RNFL. Previous studies have used PS-OCT to measure the RNFL birefringence and its distribution around the optic nerve head (ONH) in the healthy human eye. A characteristic pattern, showing higher birefringence in the superior and inferior quadrants than nasally and temporally to the ONH, has been reported [28–30,37,38]. Only very few preliminary studies on the birefringence of the RNFL in glaucomatous eyes have been reported in peer reviewed journals, providing hints for a reduced birefringence in parts of the tissue [37,39]. RNFL birefringence in patients with DR would be of great interest for the discussion of whether vascular or neural changes occur earlier in the onset of this disease.

In this pilot study, we used a state-of-the-art spectral domain PS-OCT system with an integrated retinal tracker to measure thickness, retardation, and birefringence of the RNFL in human eyes. 3D raster scans, as well as high-quality circumpapillary scans, generated by averaging 50 scans taken at the same position and stabilized by the retinal tracker, were recorded. Two methods for birefringence quantification were evaluated, and the precision of the methods was analyzed by repeated measurements in healthy eyes. To evaluate the potential of RNFL parameters for ocular diagnostics, measurements were performed in patients with early glaucoma, patients with early to moderate DR, and in age matched healthy controls. Specifically, the birefringence as an optical tissue property that is not directly accessible in vivo by other means than PS-OCT was analyzed, and differences between groups were checked for statistical significance.

## 2. Methods

### 2.1 Subjects selection

Both studies were approved by the university’s ethics committee and are in agreement with the tenets of the Declaration of Helsinki.

#### ***Repeatability study***

Five eyes of five healthy subjects were imaged. The volunteers have an average age of 35 years ((min, max) =  (28,46) years). For each volunteer, five measurements were taken with repositioning (the subject steps away from the setup), realignment (laterally and axially) and reselection of the LSLO template and circular scan pattern (as described in 2.2) in between each acquisition.

#### ***Comparative study***

Three different groups of volunteers were included in this study: healthy subjects, diabetic and glaucoma patients. Seven eyes of seven healthy subjects, seven eyes of seven diabetic patients and six eyes of six glaucoma patients were imaged. The three subsets of volunteers are age matched, with an average age of 61 years ((min, max) = (52, 74) years) for the healthy group, 64 years (min, max) = (50, 79) years) for the diabetics and 61 years ((min, max) = (53, 67) years) for the glaucoma group. The diabetic patients included in the study all had type 2 diabetes with a mild or moderate DR (diagnosed as mild if only microaneurysms are seen and moderate in presence of hemorrhages). The glaucoma patients all had early glaucoma (diagnosis was made based on the glaucomatous appearance of the optic nerve head (ONH) and a mean deviation of -6 dB or better on the visual field test). Prior to PS-OCT imaging, peripapillary RNFL thickness measurements were performed using a Spectralis OCT (Heidelberg Engineering). All imaged healthy and diabetic subjects have a RNFL thickness in the normal range as defined by the Spectralis software (version 1.10.4.0). Since the goal of this study was identification of early RNFL birefringence changes in otherwise normally appearing tissue, only glaucoma patients with an overall RNFL thickness in the normal range are included in the present study (i.e. six patients out of a set of thirty patients, imaged in the frame of a larger study). These early glaucoma patients may nevertheless already show signs of RNFL thinning in some localized areas. The exclusion of thin RNFL locations for the quantitative analysis is detailed in section 2.3.

### 2.2 Measurement protocol

A custom built spectral domain (SD) PS-OCT system with an integrated retinal tracker was used for imaging [40]. The tracking method was modified to a GPU-based algorithm in order to simplify the acquisition procedure and improve the overall reliability of the tracker. The OCT subsystem is based on a Michelson interferometer with polarization maintaining (PM) fibers. The OCT sampling beam is circularly polarized, and a polarization sensitive two-channel detection unit is used in the detection arm of the interferometer. A superluminescent diode (SLD) with a center wavelength of 860 nm and a bandwidth of 60 nm is used for imaging at an A-scan rate of 70 kHz. The resolution achieved is 4.2 µm axially (in tissue) and 17 µm laterally (the axial resolution has been determined with a mirror as the sample and measuring the full width at half maximum of the axial point spread function while the lateral resolution corresponds to the theoretical 1/e^2^ intensity diameter of the focused optical beam spot on the retina [41]). The sensitivity of the system is 98 dB. A line scanning laser ophthalmoscope (LSLO) is used for retinal tracking in real time and operates with a laser light source at a center wavelength of 786 nm. The power to the eye is 0.7 mW from the LSLO and 0.5 mW from the OCT beam, which satisfies the laser safety regulations.

Two types of scans were acquired for each subject. A raster scan consisting of 250 B-scans each containing 1024 A-scans was taken at a position including the ONH (≈ 4.5 sec per acquisition). It covers a region of 28° (x) by 21° (y) on the retina. The second type is a circular scan around the ONH at a radius of 1.5 mm (equal to the center radius of the annular region analysed by the commercial SLP instrument GDx-VCC of Carl Zeiss Meditec). The center of the ONH was manually selected on the template LSLO image used for tracking during acquisition. 100 circular B-scans each consisting of 2048 A-scans were acquired at the same position, which is maintained during the acquisition procedure by the real time tracker (≈ 3 sec per acquisition). The obtained images can be divided in four quadrants (temporal (T), superior (S), nasal (N), inferior (I)) in order to perform a TSNIT curve evaluation.

### 2.3 Data evaluation

**Fig. 1.**
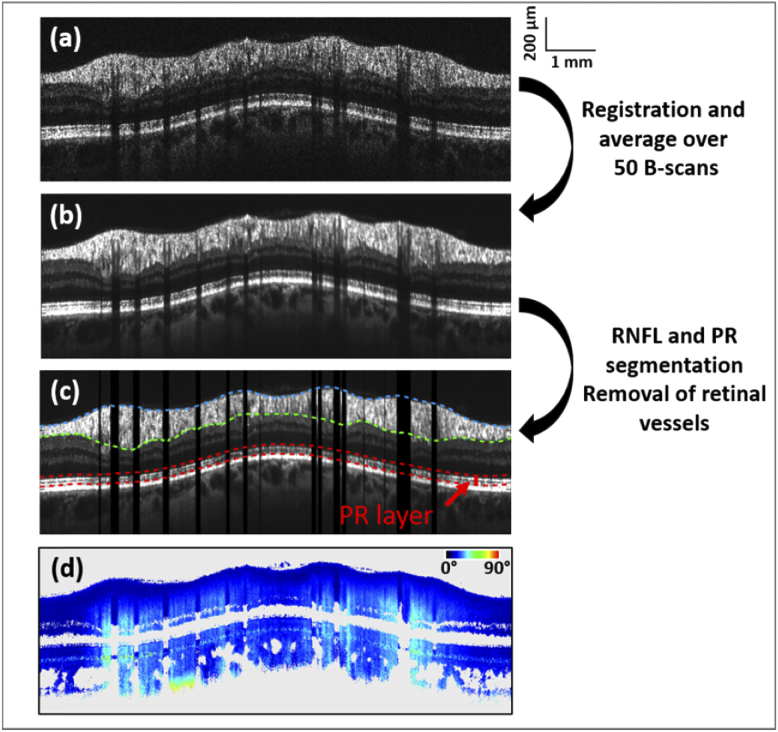
Representative circular B-scans associated with the analysis of RNFL birefringence. Averaged intensity scan (b) is obtained by registration and averaging of 50 individual B-scans (a). RNFL thickness is obtained from the segmented upper (blue) and lower (green) boundaries of the RNFL layer as shown in (c). After segmentation of the RPE, the retardation values at the photoreceptor layer (boundaries in red in (c)) are obtained from the averaged retardation scan (d) for the RNFL birefringence calculation using the quotient method. In the case of the linear regression method, the linear fit at each A-scan location, whose slope corresponds to the RNFL birefringence, also uses the averaged retardation scan (d).

After standard Fourier domain OCT processing of the data, extraction of the polarization parameters, i.e. the axis orientation and the retardation, is done for each individual B-scan [42]. B-scans compensated for anterior segment birefringence are then obtained, using an algorithm described previously [43]. The birefringence of the RNFL is derived from the retardation and thickness data and can be obtained from two different methods, as described below. For both methods, averaged circular scans of intensity and retardation are calculated as a first step. For this purpose, the 50 best correlated (to a predefined reference scan) intensity B-scans of a single acquisition sequence are chosen (Fig. 1(a)). Registration and averaging of both the intensity and retardation B-scans (cf. Figs. 1(b) and 1(d)) are then carried out, using a method based on Stokes vector averaging, as detailed in [30].

#### ***First method: The quotient method***

The RNFL birefringence value for each A-scan is calculated by:

(1) $$Birefringence\;\;({}^{\circ}/\mu m)\;\;=\;\frac{\delta_{PR}({}^{\circ})}{Thickness_{RNFL}(\mu m)}$$

Here, δ_PR_ is the retardation value extracted at the photoreceptor layer (PR). With two bands of strong signal (the boundary between inner and outer PR segments and the cone/rod outer segment tips), this layer provides the best signal to noise ratio of all the retinal layers behind the RNFL and therefore is best suited to estimate the retardation caused by the RNFL (in the vicinity of the ONH, the other layers between the RNFL and the PR are non-birefringent and don’t change the retardation). After segmentation of the retinal pigment epithelium (RPE) based on its depolarizing property [44], a band containing the PR layer is extracted, consisting of 30 pixels above the RPE (taken along each A-scan), cf. Fig. 1(c). The retardation value of each pixel is weighted by its intensity value and a histogram of all values is plotted. The peak of this histogram for a given A-scan position is taken as δ_PR_ value. The RNFL is segmented from the averaged circular intensity images as shown in Fig. 1(c) in blue and green lines. The upper boundary of the RNFL is obtained after intensity thresholding. The lower boundary of the RNFL is retrieved by a graph-search based method [45] and manual adjustments at locations of segmentation inaccuracies (mainly around the vessels and in areas where the layer is thin) were performed.

#### ***Second method: The linear regression method***

After segmentation of the RNFL (as described above for the quotient method), the RNFL retardation values are plotted against depth at each A-scan location. The corresponding RNFL birefringence value is defined as the slope of the linear regression fit of the data. As the retardation values close to the RNFL upper boundary can have a slight retardation offset, only the last two thirds of the points are used for the linear fit.

For both methods, vessel locations, corresponding to A-scan locations of low RPE intensity (caused by vessel shadows), are removed from the calculation. The RPE intensity threshold can be manually adjusted depending on the image intensity values. To avoid errors in areas of low RNFL thickness, only locations of RNFL thickness > 100 µm are taken into account. The threshold value of 100 µm was chosen based on empirical considerations: the higher the threshold, the more robust is the birefringence calculation for a single A-scan (avoiding division by a thin RNFL thickness, cf. Eq. (1), or having more data points for calculating the linear fit), while a lower threshold provides a larger number of evaluable A-scans along the circumpapillary scan. 100 µm turned out to be a good compromise for this trade-off, providing a minimum of 200 A-scans (out of the 2048 A-scans of the circular scan) for all subjects. Both methods were evaluated in the repeatability study. In the comparative study, the linear regression method was used for RNFL birefringence calculation. The choice of the method is further explained in the results (3.1) and in the discussion chapter (4). To investigate the influence of the RNFL thickness threshold on the outcome of the study, averaged birefringence results of the comparative study were additionally calculated using a 75 µm and a 125 µm threshold value (in Table 3, all other given quantitative results use the 100 µm threshold only).

From the raster scans, *en-face* maps are obtained after similar processing steps. *En-face* thickness maps are obtained after segmentation of the RNFL thickness on each individual B-scan. Finally, the *en-face* birefringence maps are then either calculated from the quotient of retardation and thickness or the linear regression fit on the RNFL retardation values at each position. The *en-face* maps shown in Fig. 3 are given for the linear regression method at locations of RNFL thickness > 75 µm (lower threshold for visualisation purpose).

## 3. Results

### 3.1 Repeatability study

Before performing a quantitative analysis of the birefringence values in the comparative study, a repeatability study was conducted on five healthy volunteers. The results are given in Table 1 for both of the calculation methods (in blue: quotient method, in orange: linear regression method). For each subject, the mean RNFL birefringence values along with the averaged RNFL thickness of circumpapillary scans are given with their corresponding standard deviation over five repetitive measurements. The two processing methods show a good repeatability with an averaged standard deviation of 3.8 µm for the thickness values and 0.006 °/µm and 0.005 °/µm for the birefringence ones, respectively, for the quotient and the linear regression methods. The RNFL birefringence values obtained from the two methods are similar, with an average difference of 0.007 °/µm. The largest difference between the two methods is about 0.011 °/µm (first subject). The repeatability being slightly better with the slope method, which is also known to be less sensitive to RNFL thickness segmentation and retardation errors [28,46,47], it was chosen for the analysis of the comparative study.

**Table 1.** Results of the repeatability study for circumpapillary scans of the RNFL. For each of the five healthy subjects, five measurements were repeated after realignment. The thickness values are calculated from the entire circumpapillary scan while the retardation and birefringence values only consider areas of RNFL thickness > 100 µm. (Note that the birefringence values can therefore not directly be calculated from the thickness and retardation values given in the table). The results of the quotient method are given in blue boxes, the ones of the linear regression in orange boxes.

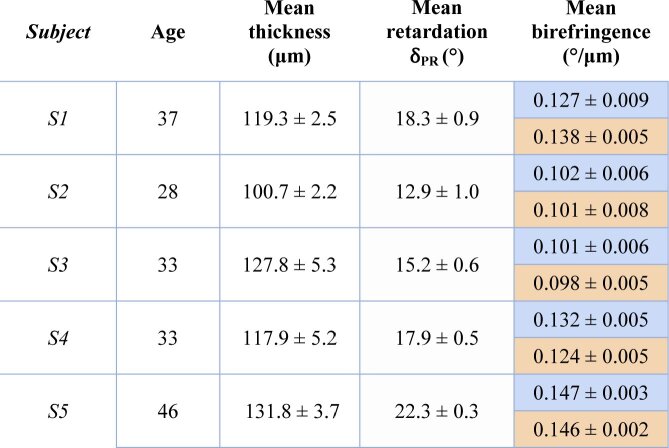

### 3.2 Comparative study: qualitative assessment

**Fig. 2.**
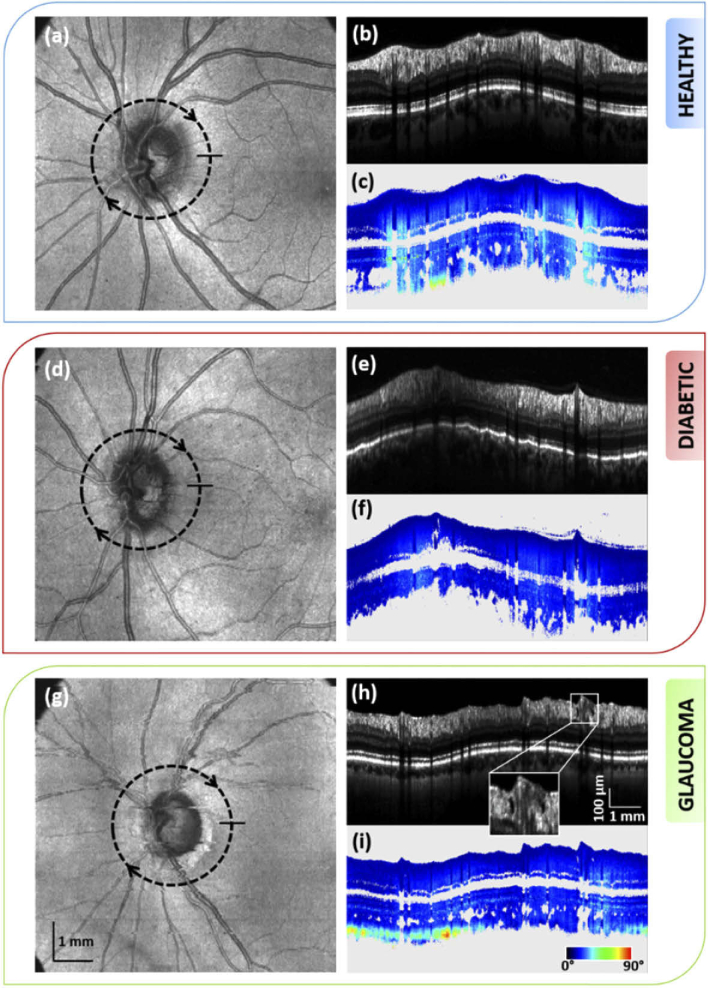
Comparison between the intensity and retardation averaged circular scan of the left eye of a healthy subject (b)-(c), a diabetic patient (e)-(f) and a glaucoma patient (h)-(i). Lower retardation is observed in the RNFL of the diseased retina as compared to the healthy one. Location of the circular scan is indicated on the corresponding *en-face* intensity maps, (a), (d) and (g). The framed area in the averaged intensity scan of the glaucoma subject (h) point out holes in the RNFL of this patient.

A comparison between the intensity and retardation averaged circular scans around the ONH of a healthy volunteer, a diabetic patient (diagnosed with moderate DR) and a glaucoma patient (early glaucoma) is given in Fig. 2. The average RNFL thickness along the circular scans is equal for the healthy (124.7 µm) and DR (124.4 µm) case, and slightly lower for the glaucoma case (109.2 µm). Despite the equal thickness, the averaged RNFL retardation of the diabetic patient (Fig. 2(f)), is considerably lower as compared to that of the healthy subject (Fig. 2(c)). No damage is visible in the appearance of the RNFL of the diabetic patient on the averaged intensity scan. In case of the glaucoma patient, the retardation (Fig. 2(i)) appears lower as compared to the healthy case. At early glaucoma stage, thinning or defects in the RNFL may already have occurred. On the averaged intensity scan of the glaucoma patient (Fig. 2(h)), holes in the RNFL can be identified in the superior sector (zoomed image of the framed area), as similarly observed in previously published work [48]. Similarly, thinning at localised areas and/or defects can be seen in three of the five additional glaucoma datasets. Such changes are not observable on any of the healthy and diabetic averaged intensity scans. These deformations, affecting the whole structure of the RNFL, may consequently impact its birefringence. As the RNFL of the diabetic patients appears similar to the ones of the heathy subjects in intensity scans, the reduced retardation should have a different origin.

**Fig. 3.**
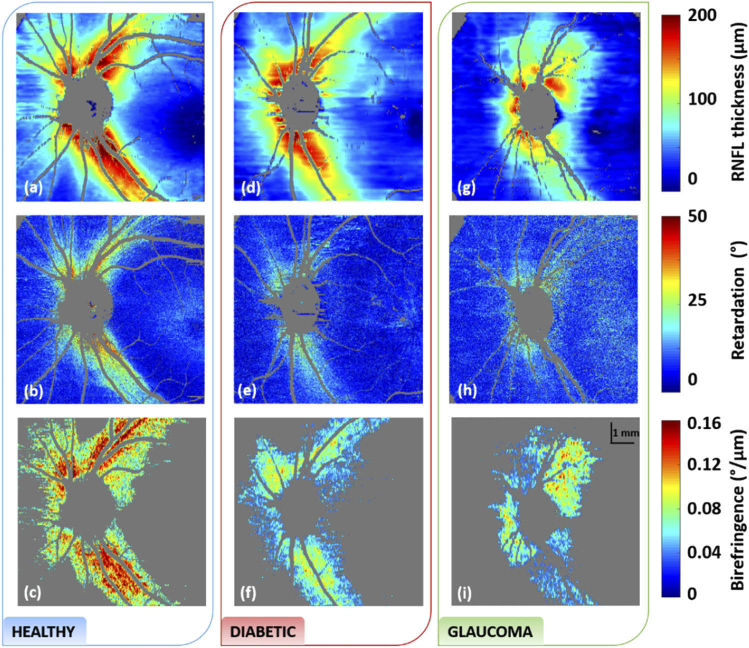
RNFL thickness (a,d,g), retardation (b, e, h) and birefringence (c, f, i) maps of the healthy, diabetic and glaucoma subjects shown in Fig. 2. Reduced retardation is seen in both the diabetic and glaucoma cases as compared to the healthy one. Reduced birefringence is particularly seen in the diabetic case, even in areas of thick NFL. A RNFL thickness threshold of 75 µm was used for visualization of the birefringence maps.

The RNFL birefringence maps of the three subjects shown in Fig. 2, along with their corresponding thickness and retardation maps are given in Fig. 3. The RNFL thickness appears reduced in the glaucoma case (Fig. 3(g)) as compared to the healthy and diabetic ones (Figs. 3(a), 3(d)). On the retardation *en-face* map of the healthy subject (Fig. 3(b)) we clearly observed a higher retardation both in the superior and inferior hemispheres, caused by the thick nerve fiber bundles in these areas. The retardation of Henle’s fiber layer, recognizable by its annular shape [49], is also seen in the foveal area. Similar results are obtained for all healthy subjects. On the retardation *en-face* map of the diabetic patient, as seen in Fig. 3(e), we observed a reduced retardation in both the superior and inferior RNFL bundles as compared to the healthy case, similarly to the results observed on the circular scan. While higher retardation values in the RNFL are still seen close to the ONH, this effect is vanishing at increasing distance. When comparing the previous images with the ones obtained for a glaucoma patient, a reduced retardation in the RNFL is similarly seen on the *en-face* retardation map in Fig. 3(h). On the birefringence maps only values where the RNFL thickness is larger than 75 µm are displayed (other areas are shown in gray). In Fig. 3(c) the birefringence values of the RNFL of the healthy subject are mostly above 0.13 °/µm in the superior and inferior sectors. In the diabetic case shown in Fig. 3(f) the birefringence values of the RNFL appear significantly reduced, most of them being around 0.10 °/µm. In the case of the glaucoma patient in Fig. 3(i), the RNFL birefringence also appears lower as compared to the values of the healthy subject. (It should be mentioned here that the en-face images, derived from a single data set each, are only used for demonstration purposes, visibly illustrating the differences between healthy and diseased cases. The rather coarse color scale does not allow for a full quantitative evaluation and differentiation between the different diseases, this is carried out on the more reliable averaged circular scans, as explained in more detail in the discussion section.)

### 3.3 Comparative study: quantitative assessment

**Fig. 4.**
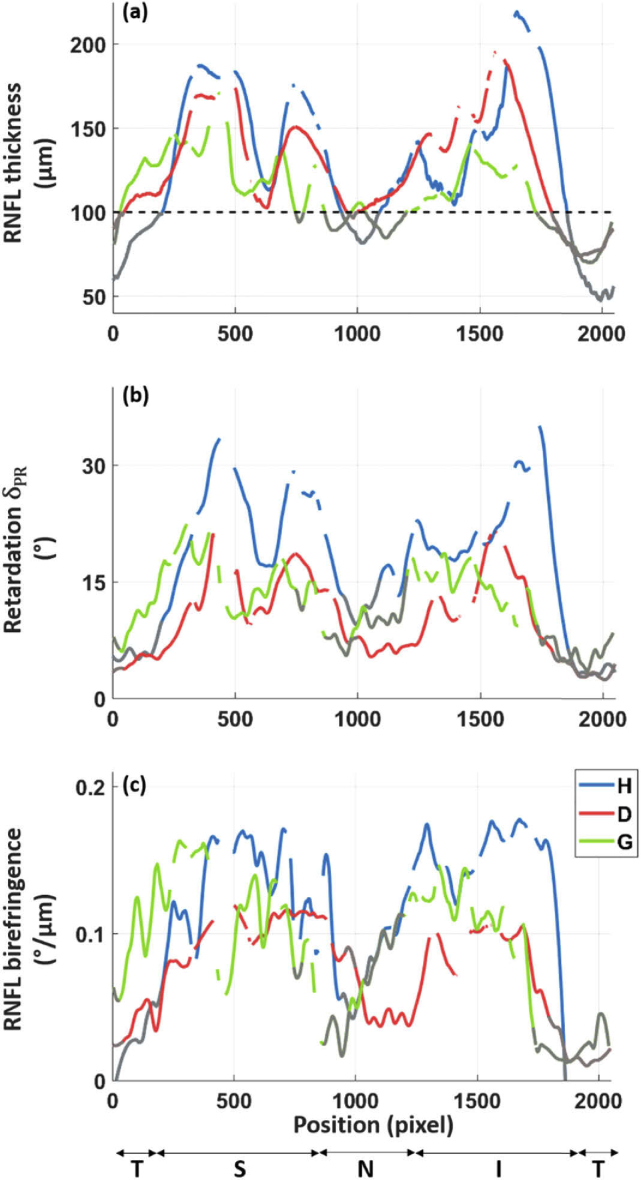
Graphs of the RNFL thickness (a), retardation (b) and birefringence (c) along a circle around the ONH for the healthy (H), diabetic (D) and glaucoma (G) subjects shown in Fig. 2. A sliding average over 10 A-scans was applied for all plots. The retardation and birefringence data were smoothen based on a local regression method. The gaps in the plots indicate vessel positions. The known double hump patterns of the RNFL are recognizable in the different graphs. The retardation and birefringence plots of the diabetic patient appear clearly lower compared to the ones of the healthy subject. While all values along the circumpapillary scan are shown here, only locations of RNFL thickness > 100 µm are used for the quantitative analysis. The values at RNFL thickness < 100 µm are displayed in gray. (T: temporal, S: superior, N: nasal, I: inferior, defined according to the GDx-VCC quadrant division).

Quantitative plots of the RNFL thickness, retardation and birefringence along the circular scans of the three subjects shown in Fig. 2 are given in Fig. 4. Similar double hump patterns of the RNFL thickness can be observed for the healthy subject and the diabetic patient (Fig. 4(a). This pattern appears a bit more flattened in the case of the glaucoma patient (Fig. 4(a)). The RNFL thickness is larger than 100 µm at a sufficient amount of A-scan locations (more than 200) for calculation of the averaged birefringence values. These locations are the ones above the dashed black line on Fig. 4(a), all other thinner areas (i.e. below this line) are not included in the quantitative birefringence analysis.

As the retardation values are proportional to the thickness of the RNFL, a similar behavior is observed for the thickness and retardation graphs for the three subjects. The averaged retardation values at the PR are considerably different for the healthy (20.9 °) and the diabetic (10.9 °) cases. For the glaucoma patient, the averaged retardation from the circular scan is 14.1°. Despite its thinner RNFL, as compared to the diabetic case, the glaucoma patient has a higher averaged retardation value.

Finally, the birefringence, as the parameter that relates retardation and thickness, is calculated. The birefringence distribution along the circular scan of the healthy subject appears higher along the superior and inferior parts of the RNFL as compared to the birefringence distributions of the diabetic and glaucoma patients (Fig. 4(c)). The calculated means of the birefringence are 0.134°/µm, 0.078°/µm and 0.114°/µm, respectively, for the healthy volunteer, the diabetic patient and the glaucoma patient.

**Fig. 5.**
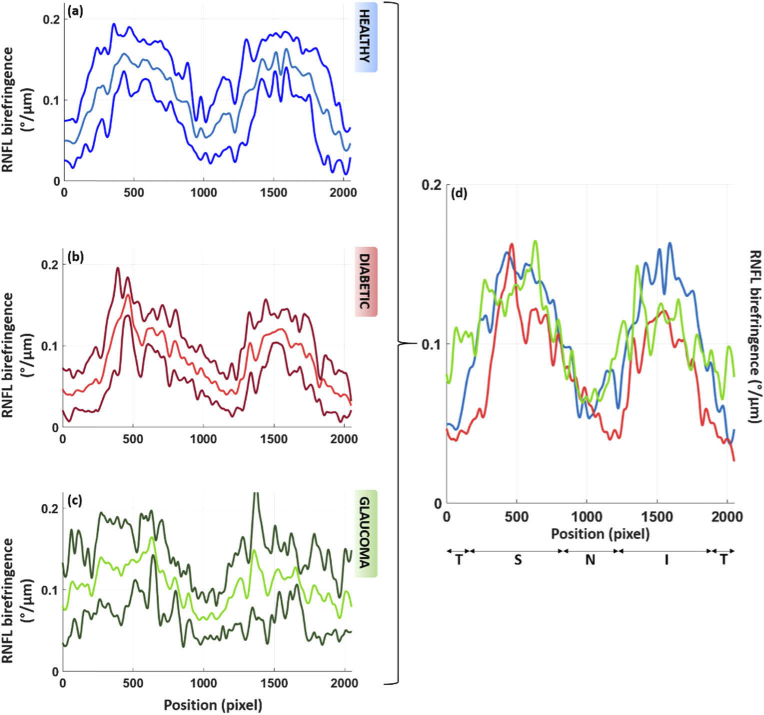
Averaged RNFL birefringence along the circular scan for the three groups of volunteers, respectively, for the healthy subjects (a), the diabetic patients (b) and the glaucoma patients (c). The central line shows the mean value over the subjects of each group. The other lines show ± one standard deviation. To ease the comparison, the mean plots of each group are overlapped in (d). The averaged values are lower for the diabetic and glaucoma patients as compared to the healthy subjects. While all values along the circumpapillary scan are shown here, only locations of RNFL thickness > 100 µm are used for the quantitative analysis. Similarly to Fig. 4, (a) sliding average over 10 A-scans and smoothing of the data were applied for all plots.

The mean distribution (averaged over all subjects in each group) of the RNFL birefringence values along the circular scan and its standard deviation are given for each subject group in Fig. 5. The values reached in the double hump patterns are lower for the diabetic and glaucoma patients as compared to the heathy subjects, especially in the inferior sector. While the overall profile of the curves (despite the difference in values) of the healthy and diabetic set appear similar, the pattern seems more irregular for the glaucoma group. This might be linked to the localized defects which can be seen in the RNFL of the glaucoma patients.

Table 2 summarizes the averaged circumpapillary thickness and birefringence values over the entire set of data of this study. All subjects have a mean RNFL thickness in the normal range (as compared to the values found in the literature [50–52]) with an averaged value at a radius 1.5 mm away from the ONH ranging from 84 µm to 133.4 µm for the healthy subjects, 96.2 µm to 124.4 µm for the diabetic patients and 101.7 µm to 120.7 µm for the glaucoma patients (patient inclusion criteria require a RNFL thickness > 100 µm in at least 200 A-scans out of the 2048 A-scans). The averaged mean birefringence values in the RNFL of the healthy volunteers is 0.135 ± 0.007 °/µm. The averaged mean birefringence values are lower than in the healthy case both for the diabetic and glaucoma cases. The values are particularly low for the diabetic patients, with a mean birefringence of 0.103 ± 0.015 °/µm, while the mean birefringence is 0.129 ± 0.013 °/µm for the glaucoma patients. The reduced birefringence observed in the diabetic patients (as compared to the healthy ones) is statistically highly significant (t-test, two tailed) with a p-value of 7·10^−4^. This is not the case for the glaucoma patients, where the p-value is about 0.4 (when compared to the healthy subjects).

**Table 2.** Averaged RNFL thickness, retardation and birefringence values for the set of heathy volunteers (n = 7), diabetic (n = 7) and glaucoma patients (n = 6). The thickness values are calculated from the entire circumpapillary scan while the retardation and birefringence values only consider areas of RNFL thickness > 100 µm (excluding, for example, areas of possible RNFL thinning in patients). For each parameter, the p-values of the t-test between healthy and diabetic and healthy and glaucoma are given. A statistically significantly reduced birefringence is seen in the RNFL of diabetic patients as compared to the heathy subjects (p=7·10^−4^).

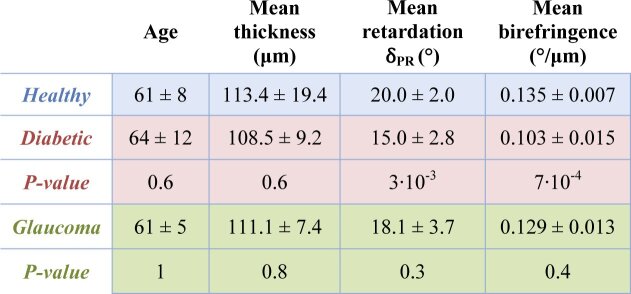

Table 3 compares the averaged RNFL birefringence values of the three groups using different RNFL thickness thresholds, i.e. 75 µm, 100 µm (the reference cut-off of Table 2) and 125 µm. With increasing threshold, there is a slight increase in mean birefringence. The reason for this is likely that lower thresholds lead to the inclusion of a larger number of A-scans in the nasal and temporal sectors, which are known to have lower birefringence [29,30]. However, the significance of the statistical results is similar for the three tested threshold values.

**Table 3.** Averaged RNFL birefringence values using different RNFL thickness thresholds for the same set of healthy volunteers, diabetic and glaucoma patients as used in Table 2. Areas of RNFL thickness > 75 µm, RNFL thickness > 100 µm (values of Table 2) and RNFL thickness > 125 µm are considered, respectively, in the three different columns. The statistically significantly reduced birefringence in the RNFL of diabetic patients as compared to the heathy subjects is seen for all of the three threshold values tested.

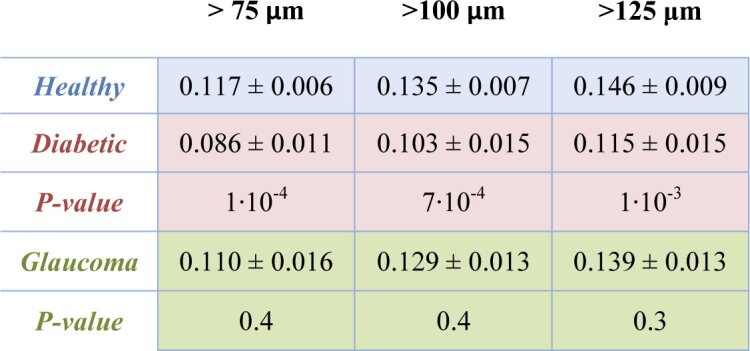

## 4. Discussion

In this pilot study, we used a custom developed, state of the art PS-OCT instrument with an integrated retinal tracker to measure the birefringence of the RNFL in the region around the ONH of healthy subjects, patients with early glaucoma, and diabetics with early to moderate DR. Quantitative measurements of the birefringence were derived from high-quality averaged circumpapillary scans.

In a first step, we analyzed the reproducibility (precision) of two different birefringence evaluation methods in healthy eyes over five repetitive measurements, with realignment and reselection of the template for tracking in between each. We obtained a standard deviation of 0.006°/µm and 0.005°/µm for the birefringence, averaged over the locations of the circumpapillary scan of RNFL thickness >100 µm, respectively, for the quotient and the linear regression methods. These values are within the range of values previously reported for measurements obtained for circumpapillary scans derived from 3D data sets (where the linear regression method was used) [29]. The residual variations might be caused by the selection of the center point of the circumpapillary scans, which is presently done manually and might lead to slight variations of the scan trace from scan to scan, yielding slight variations in RNFL thickness and birefringence values. In order to further increase the repeatability results, an automatic detection of the ONH center could be implemented. It should be mentioned here that, while the reproducibility of the method is good, the absolute accuracy would be very difficult to estimate: the only method that works in vivo would be the use of thickness and retardation values obtained by separate instruments (OCT and SLP), which, however, would be even less reliable, given the known sensibility of SLP to artifacts caused by other birefringent tissues in the eye, and by the necessity of image registration. Ex-vivo measurements of RNFL birefringence, impossible in human subjects, could only be done in animal models but would suffer from possible change of tissue optical properties during tissue preparation. However, as discussed below, the absolute birefringence values reported here are in the range reported earlier by measurements with other PS-OCT instruments.

**Fig. 6.**
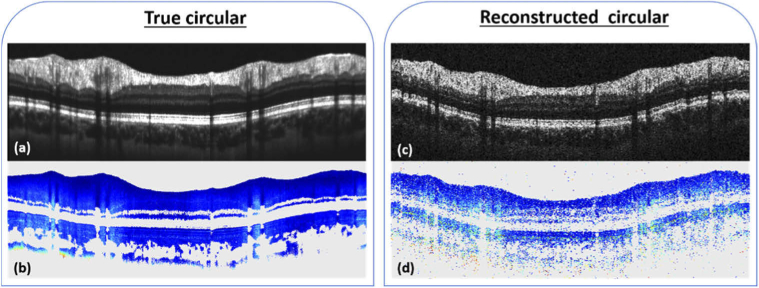
Comparison between the directly recorded (averaged over 50 B-scans) circumpapillary scans - intensity (a) and retardation (b) – and images reconstructed from a 3D data set along the same circle as for the circular scan – intensity (c) and retardation (d) – of a healthy subject.

The recording of circumpapillary scans has the advantage to retrieve highly averaged intensity and retardation tomograms with a similar acquisition time as for the reconstructed circumpapillary scans obtained from 3D data sets. The scanning position is maintained during acquisition using the retinal tracker. Compensating for eye saccades and large movements may however not be possible for all of the recorded scans, which could lead to a decrease of the overall data quality. Therefore only the 50 best correlated circular B-scans (from the 100 recorded per acquisition) are registered and averaged for the birefringence calculation. Lower quality scans are neglected, limiting the impact of the acquisition inaccuracies. The resulting averaged scans are of high quality and provide an excellent base for segmentation of the RNFL (intensity data) and for extracting retardation and birefringence from low-noise polarization data. In principle, the same type of circumpapillary data evaluation could be extracted from 3D raster scan data. However, these data are much noisier (because they are not averaged) and are reconstructed from unequally sampled data (in x and y direction), making the data less reliable. For illustration purposes, Fig. 6 shows circumpapillary data sets obtained by direct circular scanning and averaging (cf. Figs. 6(a), 6(b)) and by extraction from a 3D data set (cf. Fig. 6(c), 6(d)). The main limitation of the latter method is that the 3D scans record large amounts of data in areas outside the analyzed circle that are later discarded, while the circular scans are restricted to the evaluation position. Thus, for a similar recording time way more measurement points (A-scans) are acquired in the region of interest. This is especially important for patient data that frequently suffer from poor signal quality and stronger motion artifacts.

In a second step, we measured the RNFL birefringence in healthy and diseased eyes using the linear regression method as it provides a slightly higher precision and allows for a better comparison with previously reported results that also used this method. In healthy eyes, we obtained birefringence results of the order of 0.135 °/µm within RNFL tissue of thickness > 100 µm. This is within the range of values previously reported [28,29,37,38]. For areas of thin RNFL, such as the temporal and nasal regions, the results would require a more careful analysis. An accurate linear regression fit may not be possible as the RNFL only covers a few data points in depth. In such regions, the quotient method may be more precise despite being still highly dependent on the noise of the acquired data. Moreover, the quotient method is strongly dependent on an accurate segmentation of the RNFL thickness. This is particularly critical in thin RNFL regions, where a difference of a few pixels will greatly affect the birefringence calculation, and at the vicinity of retinal vessels, where shadowing might lead to regions of apparently lower birefringence. In case of segmentation failure, the segmented lines were manually adjusted for this study. The vessel locations were excluded based on the position of vessel shadows on the RPE on the averaged intensity scans. The birefringence analysis in the thinner temporal and nasal areas could provide additional diagnostic information, however, would require further refinements of post-processing steps and is beyond the scope of this study.

Only very preliminary PS-OCT studies on the RNFL birefringence of glaucomatous eyes have yet been published in peer reviewed journals [37,39]. They observed hints of reduced birefringence, however, data were obtained only from one glaucomatous eye, each, and data suffered from noise. Our pilot study covers six eyes with early stage glaucoma and compares the results to age-matched controls. We observed a reduced RNFL birefringence of 0.129 °/µm. This observation of reduced birefringence is in agreement with results obtained by a combination of OCT and scanning laser polarimetry in experimental glaucoma in non-human primates [31,53]. This reduction might be caused by a change in density of ganglion cell axons or their microtubules, and it has been speculated that ganglion cell axon density changes before a reduction of RNFL thickness can be observed [32]. Therefore, a reduced RNFL birefringence might be an early indicator of glaucomatous damage (although a larger study would have to show if this observation is significant).

To the best of our knowledge, no study of RNFL birefringence of patients with DR has yet been reported. While some reduction of birefringence in the glaucoma patients had been expected, the even stronger reduction observed in the DR patient group (0.103 °/µm) came as a surprise. This reduction is statistically highly significant (p = 7 · 10^−4^ for the birefringence value averaged along circumpapillary scans). Contrary to the glaucomatous cases, the RNFL seems to be a continuous and smooth tissue in the intensity scans of the DR eyes, without tiny holes or other visible deformation or damage. So we can only speculate as to the reasons for this birefringence reduction. It might be caused by some structural change of RGC axons and/or their microtubules on a very small scale. More detailed histopathology studies might be needed for a better understanding of this effect. It should be mentioned here that in the first part of the study (repeatability study in 3.1) two healthy subjects show lower RNFL birefringence (S2 and S3 with mean values of respectively 0.101 °/µm and 0.098 °/µm using the linear regression method) while this was not the case in the comparative study (lowest value obtained is 0.124 °/µm). Since these subjects are not age matched with the diabetic and glaucoma patients, they were not used for comparison. It however gives a hint about the possible influence of the age on the RNFL birefringence, which would need to be further investigated in a larger study. In addition, studies in a larger number of patients with different stages of DR, as well as in diabetic patients without DR, might provide a better understanding. This may finally help to answer the question whether microvascular or neurologic changes occur first in the onset of the disease and might turn out to provide a very useful diagnostic tool.

## 5. Conclusion

In this work we presented a quantitative comparison of the RNFL birefringence (at RNFL thickness >100 µm) in a small number of healthy subjects, early glaucoma and diabetic patients with mild to moderate DR using a PS-OCT system. The reproducibility of two calculation methods was first tested on healthy subjects and the more precise one was chosen for the comparative analysis. The results show a reduced birefringence in the RNFL of glaucoma patients, however not statistically significant. A further reduced birefringence that was statistically highly significant (as compared to the healthy group) was observed in the RNFL of the diabetic patients. While defects can be seen in the RNFL of the glaucoma patients, no damages can be seen in the RNFL of the diabetic patients. A better understanding of the underlying mechanisms, such as potential structural change of RGC axons, would require a larger scale study and comparison to histopathological data.
